# Supplementary material for: Gene-centric functional dissection of human genetic variation uncovers regulators of hematopoiesis
Source: eLife. 2019 May 9;8:e44080. doi: 10.7554/eLife.44080 (PMC6534380; doi:10.7554/eLife.44080)
Supplement: Transparent reporting form [file elife-44080-transrepform.docx]

***eLife’s* transparent reporting form**

We encourage authors to provide detailed information *within their submission* to facilitate the interpretation and replication of experiments. Authors can upload supporting documentation to indicate the use of appropriate reporting guidelines for health-related research (see [EQUATOR Network](http://www.equator-network.org/%20)), life science research (see the [BioSharing Information Resource](https://biosharing.org/)), or the [ARRIVE guidelines](http://www.plosbiology.org/article/info:doi/10.1371/journal.pbio.1000412) for reporting work involving animal research. Where applicable, authors should refer to any relevant reporting standards documents in this form.

If you have any questions, please consult our Journal Policies and/or contact us: [editorial@elifesciences.org](mailto:editorial@elifesciences.org).

**Sample-size estimation**

- You should state whether an appropriate sample size was computed when the study was being designed
- You should state the statistical method of sample size computation and any required assumptions
- If no explicit power analysis was used, you should describe how you decided what sample (replicate) size (number) to use

Please outline where this information can be found within the submission (e.g., sections or figure legends), or explain why this information doesn’t apply to your submission:

Samples sizes were chosen based upon conventional practices. For the creation of the hairpin library, hairpin numbers targeting each gene were dictated by the unique hairpins available in the Broad Institute GPP archive with a goal of including at least five hairpins targeting a single gene to be able to account appropriately for off target effects and variation in on target efficiency. The sets of essential and negative control hairpins were also chosen from those available from the GPP and to allow sufficient numbers for appropriate controls. Three independent human donor CD34^+^ cell populations were used to screen the library based upon availability and common employment of triplicates in such screens performed in primary cells.

**Replicates**

- You should report how often each experiment was performed
- You should include a definition of biological versus technical replication
- The data obtained should be provided and sufficient information should be provided to indicate the number of independent biological and/or technical replicates
- If you encountered any outliers, you should describe how these were handled
- Criteria for exclusion/inclusion of data should be clearly stated
- High-throughput sequence data should be uploaded before submission, with a private link for reviewers provided (these are available from both GEO and ArrayExpress)

Please outline where this information can be found within the submission (e.g., sections or figure legends), or explain why this information doesn’t apply to your submission:

All replicate measurements reported in this study represent independent biological replicates and are discussed in the text/ figure legends or within the original cited literature from which external datasets were retrieved. In the case of our shRNA screen, primary CD34^+^ cells from three unrelated human donors were used. No data or outliers were excluded from reporting. All follow-up experiments were repeated at least three times. The number of replicates can be found in the figure legends.

**Statistical reporting**

- Statistical analysis methods should be described and justified
- Raw data should be presented in figures whenever informative to do so (typically when N per group is less than 10)
- For each experiment, you should identify the statistical tests used, exact values of N, definitions of center, methods of multiple test correction, and dispersion and precision measures (e.g., mean, median, SD, SEM, confidence intervals; and, for the major substantive results, a measure of effect size (e.g., Pearson's r, Cohen's d)
- Report exact p-values wherever possible alongside the summary statistics and 95% confidence intervals. These should be reported for all key questions and not only when the p-value is less than 0.05.

Please outline where this information can be found within the submission (e.g., sections or figure legends), or explain why this information doesn’t apply to your submission:

For all hypothesis testingm p values are reported accompanying the corresponding results and figures, and details for the statistical tests or permutations used are included in the methods. Corrections for multiple testing are also described. Measures of central tendency and variance, where portrayed, are detailed in the text and figure legends.

(For large datasets, or papers with a very large number of statistical tests, you may upload a single table file with tests, Ns, etc., with reference to sections in the manuscript.)

**Group allocation**

- Indicate how samples were allocated into experimental groups (in the case of clinical studies, please specify allocation to treatment method); if randomization was used, please also state if restricted randomization was applied
- Indicate if masking was used during group allocation, data collection and/or data analysis

Please outline where this information can be found within the submission (e.g., sections or figure legends), or explain why this information doesn’t apply to your submission:

No specific grouping allocations were used in this study.

**Additional data files (“source data”)**

- We encourage you to upload relevant additional data files, such as numerical data that are represented as a graph in a figure, or as a summary table
- Where provided, these should be in the most useful format, and they can be uploaded as “Source data” files linked to a main figure or table
- Include model definition files including the full list of parameters used
- Include code used for data analysis (e.g., R, MatLab)
- Avoid stating that data files are “available upon request”

Please indicate the figures or tables for which source data files have been provided:

Source data for the shRNA screen, along with all relevant derived quantities, have been included in the supplemental tables. Analysis code used to analyze the dataset will be uploaded to the Sankaran Laboratory GitHub repository at: https://github.com/orgs/sankaranlab.
